# Human Dermal Stem/Progenitor Cell-Derived Conditioned Medium Improves Senescent Human Dermal Fibroblasts

**DOI:** 10.3390/ijms160819027

**Published:** 2015-08-13

**Authors:** Ji-Yong Jung, Joong Hyun Shim, Hyun Choi, Tae Ryong Lee, Dong Wook Shin

**Affiliations:** Amorepacific Corporation R&D Center, 314-1 Bora-dong, Giheung-gu, Yongin-si, Geyonggi-do 446729, Korea; E-Mails: ninefog@amorepacific.com (J.-Y.J.); ungstrong@hanmail.net (J.H.S.); happycell@amorepacific.com (H.C.)

**Keywords:** fibroblast, human dermal stem/progenitor cells, senescence

## Abstract

Adult skin stem cells are recognized as potential therapeutics to rejuvenate aged skin. We previously demonstrated that human dermal stem/progenitor cells (hDSPCs) with multipotent capacity could be enriched from human dermal fibroblasts using collagen type IV. However, the effects of hDSPCs on cellular senescence remain to be elucidated. In the present study, we investigated whether conditioned medium (CM) collected from hDSPC cultures (hDSPC-CM) exhibits beneficial effects on senescent fibroblasts. We found that hDSPC-CM promoted proliferation and decreased the expression level of senescence-associated β-galactosidase in senescent fibroblasts. In addition, p53 phosphorylation and p21 expression were significantly reduced in senescent fibroblasts treated with hDSPC-CM. hDSPC-CM restored the expression levels of collagen type I, collagen type III, and tissue inhibitor of metalloproteinase, and antagonized the increase of matrix metalloproteinase 1 expression. Finally, we demonstrated that hDSPC-CM significantly reduced reactive oxygen species levels by specifically up-regulating the expression level of superoxide dismutase 2. Taken together, these data suggest that hDSPC-CM can be applied as a potential therapeutic agent for improving human aged skin.

## 1. Introduction

Normal somatic cells cultured *in vitro* display a limited capacity to divide, and thus eventually become senescent [1]. Various harmful signals, including oxidative stress, DNA damage, and telomere shortening, have been reported to promote cellular senescence [2,3]. This cellular status is characterized by growth arrest, enlarged cell size, increased expression of senescence-associated β-galactosidase (SA-β-Gal), and accumulation of the tumor suppressors p53 and p21, which participate in cell cycle arrest [4,5]. Cellular senescence is closely related to the aging process; the number of senescent cells gradually increases with age, and this accumulation contributes to tissue aging and the development of age-related diseases [6].

Adult stem cells are indispensable for maintaining homeostasis in all tissues of the body. Representative characteristics of these stem cells are their self-renewal and multipotent capacities [7,8,9,10,11,12,13,14,15,16,17,18]. These cells have been identified in various tissues, including the bone marrow [7,8,9], skeletal muscle [10], heart [11], adipose tissue [12], and skin [13,14,15,16,17,18]. We previously demonstrated that collagen type IV-enriched human dermal stem/progenitor cells (hDSPCs) exhibit stem cell-like characteristics [17]. We also recently reported that conditioned medium (CM) collected from hDSPCs (hDSPC-CM) improved human dermal fibroblasts (HDFs) damaged by ultraviolet A (UVA), which causes photoaging of the skin [19].

In the present study, we expanded on these findings and examined whether hDSPC-CM exerts beneficial effects on the cellular senescence that occurs in aging skin. We found that hDSPC-CM significantly increased proliferation, and reduced SA-β-gal expression, p53 phosphorylation, and reactive oxygen species (ROS) levels in senescent fibroblasts. These data suggest that hDSPC-CM is a potential stem cell-based therapeutic agent to prevent aging in the skin.

## 2. Results

### 2.1. hDSPC-CM Increased Cell Proliferation in Senescent Fibroblasts

**Figure 1:**
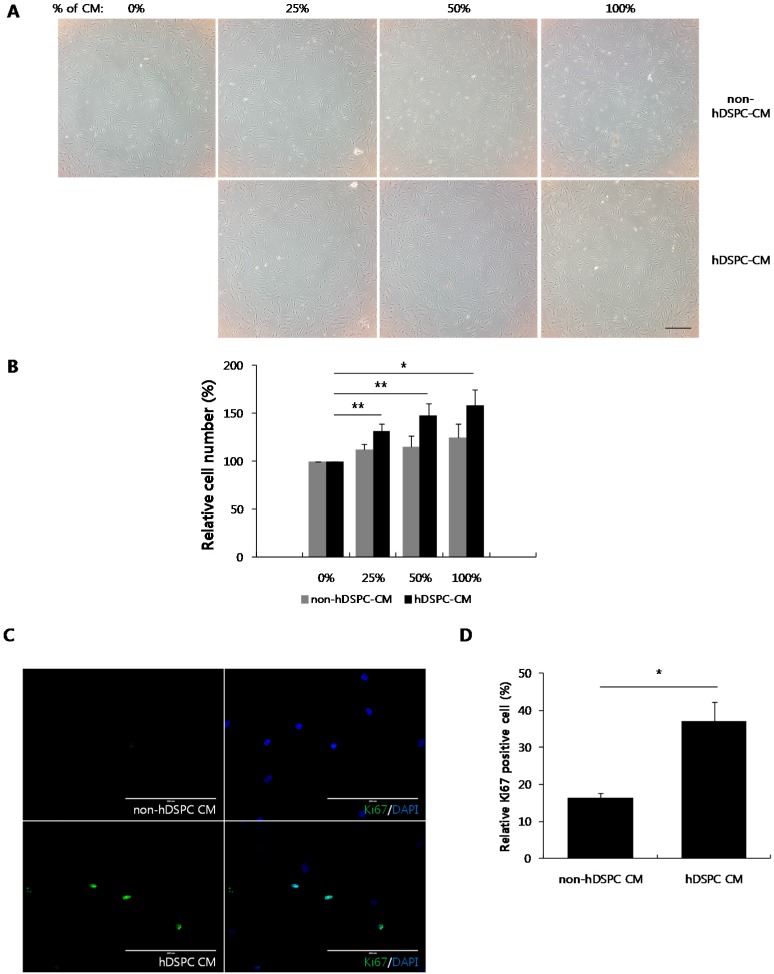
hDSPC-CM promoted proliferation in senescent fibroblasts. Senescent fibroblasts were treated with the indicated concentrations of either non-hDSPC-CM (**upper** panel) or hDSPC-CM (**lower** panel). Images were taken 24 h after CM treatment. The scale bar represents 500 μm (**A**); Senescent fibroblasts were incubated with the indicated concentrations of each CM for 24 h and cell-counting assays were performed. Graphs depict the means ± S.E.M of three independent experiments. * *p* < 0.05, ** *p* < 0.01 (**B**); Senescent fibroblasts were treated with 50% of either non-hDSPC-CM or hDSPC-CM for 24 h. Immunofluorescent staining assays were performed with anti-Ki67 (green) antibody and DAPI (blue). The scale bar represents 400 μm (**upper** panel) or 200 μm (**lower** panel) (**C**); Ki67-positive cells were quantified. The graph depicts means ± S.E.M from three independent experiments. * *p* < 0.05 (**D**). Data were statistically compared with the unpaired Student’s *t*-test (**B**,**D**).

We previously demonstrated that hDSPC-CM could restore UVA-induced damage in HDFs [19]. In the current study, we investigated whether hDSPC-CM would similarly reverse cellular senescence. First, we evaluated the effects of hDSPC-CM on the proliferation of senescent fibroblasts. We observed increased proliferation in senescent fibroblasts treated with hDSPC-CM compared with cells treated with non-hDSPC-CM (Figure 1A). A cell proliferation assay further revealed that hDSPC-CM promoted the proliferation of senescent fibroblasts in a concentration-dependent manner, whereas non-hDSPC-CM did not (Figure 1B). As expected, senescent fibroblasts expressed lower levels of the cell proliferation marker Ki67 compared with control fibroblasts (data not shown). However, treatment with hDSPC-CM, but not non-hDSPC-CM, significantly enhanced Ki67 expression in senescent fibroblasts (Figure 1C,D).

### 2.2. hDSPC-CM Inhibited Senescence-Related Signaling in Fibroblasts

**Figure 2:**
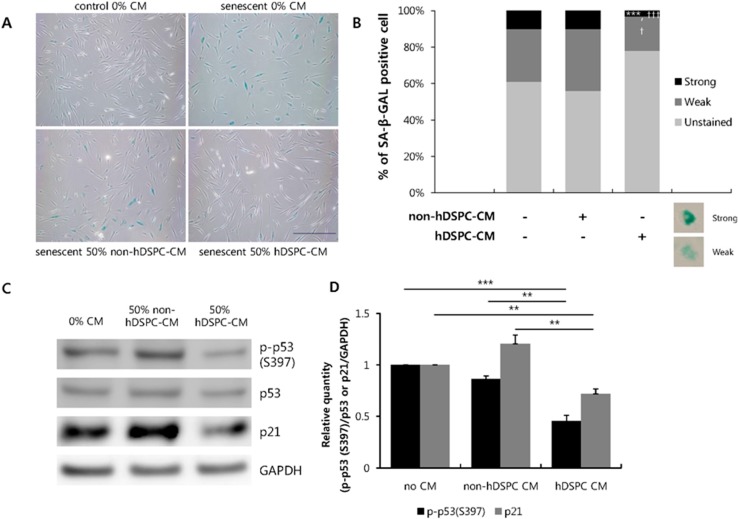
hDSPC-CM reduced the expression of senescence-associated markers in senescent fibroblasts. Senescent fibroblasts were incubated with either 0% or 50% of each CM for 72 h. SA-β-Gal-positive cells appeared blue when visualized with optical microscopy. The scale bar indicates 500 μm (**A**); Quantification of SA-β-Gal-positive cells in panel A. *** *p* < 0.001 *vs.* 0% CM control; ^†^ *p* < 0.05, ^†††^ *p* < 0.001 *vs.* non-hDSPC-CM group (**B**); Western blot analysis of p53 and p21 in each group of CM-treated senescent fibroblasts. The image represents three independent experiments and graphs depict means ± S.E.M. ** *p* < 0.01, *** *p* < 0.001 (**C**,**D**). Data were statistically compared with one-way analysis of variance (**B**) or an unpaired Student’s *t*-test (**D**).

Next, we investigated whether hDSPC-CM could reverse cellular senescence. SA-β-Gal is a well-known marker of cellular senescence in various cells, including fibroblasts [20,21]. We observed higher SA-β-Gal expression in senescent fibroblasts compared to young fibroblasts (Figure 2A, upper panel). However, hDSPC-CM treatment significantly decreased the number of SA-β-Gal-positive cells relative to fresh medium (0% CM) or non-hDSPC-CM treatment (Figure 2A, lower panel and 2B).

The tumor suppressor p53 regulates cell cycle arrest, apoptosis, and cellular senescence [2,3,4,5]. We thus investigated whether hDSPC-CM could inhibit the p53 signaling pathway. Treatment with hDSPC-CM for 72 h reduced p53 phosphorylation. The expression level of p21, which is regulated by p53, was also inhibited by hDSPC-CM treatment (Figure 2C,D). In summary, our results suggest that hDSPC-CM could contribute to reduce cellular senescence by suppressing p53 signaling.

### 2.3. hDSPC-CM Enhanced HDF-Specific Markers

**Figure 3:**
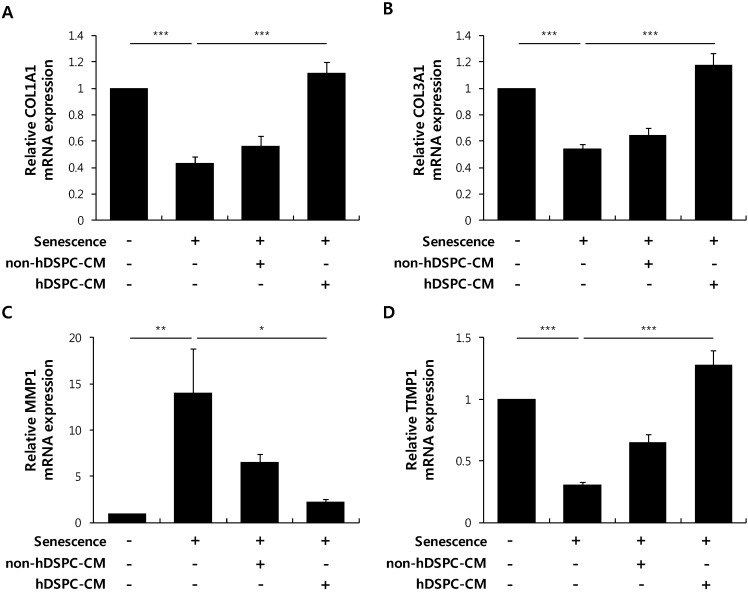
hDSPC-CM restored the expression of specific dermal makers in senescent fibroblasts. Senescent fibroblasts were treated with either hDSPC-CM or non-hDSPC-CM for 24 h. Real-time RT-PCR analysis was performed to quantify the expression levels of *COL1A1* (**A**); *COL3A1* (**B**); *MMP1* (**C**); and *TIMP1* (**D**). The graphs depict means ± S.E.M of eight independent experiments. * *p* < 0.05, ** *p* < 0.01, *** *p* < 0.001. Data were statistically compared with one-way analysis of variance.

We next investigated whether hDSPC-CM affects the expression levels of fibroblast-specific markers in senescent fibroblasts. The transcript levels of collagen type 1 alpha 1 (*COL1A1*), collagen type 3 alpha 1 (*COL3A1*), and tissue inhibitor of metalloproteinase (*TIMP1*), which are important components of the skin dermis, were significantly reduced in senescent fibroblasts compared with normal fibroblasts, and hDSPC-CM treatment significantly increased the expression levels of all three markers (Figure 3A,B and D). Conversely, matrix metalloproteinase 1 (*MMP1*) transcripts were increased in senescent fibroblasts compared with normal fibroblasts, and hDSPC-CM treatment significantly reduced *MMP1* expression (Figure 3C).

### 2.4. hDSPC-CM Suppressed H_2_O_2_ Generation in Senescent Fibroblasts

**Figure 4:**
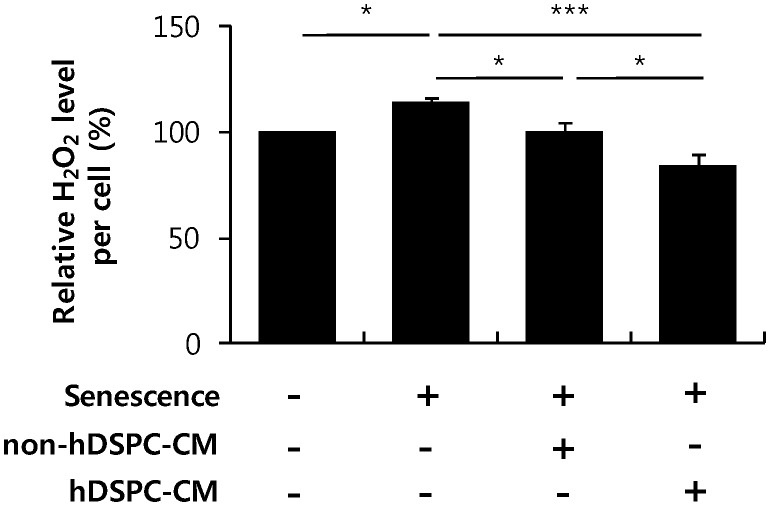
hDSPC-CM inhibited H_2_O_2_ generation in senescent fibroblasts. Basal H_2_O_2_ levels in senescent fibroblasts were measured with Amplex Red fluorescent dye at 24 h after treatment with the indicated CMs, as described in the Experimental Section. Graphs depict means ± S.E.M of three independent experiments. * *p* < 0.05, *** *p* < 0.001. Data were statistically compared with one-way analysis of variance.

Several studies have reported that increased oxidative stress causes cellular senescence and aging [22,23,24]. Therefore, we examined whether hDSPC-CM could suppress ROS generation in senescent fibroblasts. Senescent fibroblasts produced higher ROS levels than normal fibroblasts [22,23]. Although both non-hDSPC-CM and hDSPC-CM inhibited the level of H_2_O_2_ in senescent fibroblasts, hDSPC-CM treatment more significantly reduced H_2_O_2_ generation (Figure 4).

### 2.5. hDSPC-CM Specifically Increased the Superoxide Dismutase 2 (SOD2) Expression Level among ROS Scavenging Enzymes in Senescent Fibroblasts

**Figure 5:**
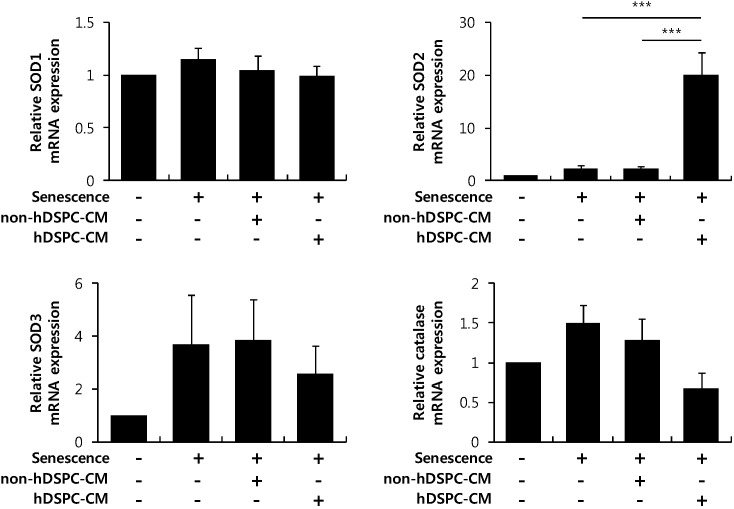
hDSPC-CM specifically up-regulated the expression level of the ROS scavenging enzyme SOD2 in senescent fibroblasts. Senescent fibroblasts were treated with either hDSPC-CM or non-hDSPC-CM for 24 h. Real-time RT-PCR was performed for *SOD1*, *SOD2*, *SOD3*, and catalase. Graphs depict means ± S.E.M of four independent experiments. *** *p* < 0.001. Data were compared with one-way analysis of variance.

ROS scavenging enzymes are responsible for reducing H_2_O_2_ levels to maintain cell homeostasis [21,22,23]. Thus, we tested whether hDSPC-CM could increase the expression of ROS scavenging enzymes such as superoxide dismutases (SODs) and catalase. Interestingly, among the ROS scavenging enzymes tested, hDSPC-CM specifically enhanced the expression level of SOD2 in senescent fibroblasts (Figure 5).

## 3. Discussion

The aging process gradually decreases the homeostatic and regenerative potential of all tissues [25,26,27,28]. In particular, photoaging and intrinsic aging decreases the elasticity of the skin, resulting in the formation of wrinkles and impairing wound healing [29,30,31]. These age-related changes might reflect the gradual increase in the proportion of senescent cells in specific tissues. Adult stem cells have been considered as potential therapeutics to slow this aging process. Indeed, we recently demonstrated that hDSPC-CM restored UVA-induced damages to fibroblasts [19].

In the present study, we further demonstrated that hDSPC-CM reversed multiple phenotypes associated with cellular senescence. First, we found that hDSPC-CM significantly enhanced the proliferation of senescent fibroblasts (Figure 1). Second, we found that hDSPC-CM significantly decreased SA-β-gal production, p53 phosphorylation, and the expression level of p21, all of which were generally increased in senescent fibroblasts (Figure 2). Third, RT-PCR analyses revealed that hDSPC-CM restored the expression of major dermal biomarkers, including *COL1A1*, *COL3A1*, and *TIMP1*, which were typically down-regulated in senescent fibroblasts (Figure 3). Fourth, we demonstrated that hDSPC-CM significantly decreased H_2_O_2_ levels by specifically increasing SOD2 expression (Figure 4 and Figure 5).

Mesenchymal stem cell (MSC) transplantation accelerates the wound-healing process in damaged skin [32,33,34]. Moreover, some MSC transplantation studies have suggested that the therapeutic potential of MSCs might be mediated by secreted growth factors rather than their long-term presence in injured tissues [35,36]. Recent studies demonstrated that CM harvested from stem cell cultures exerts beneficial effects on multiple cellular defects and diseases [37,38,39]. For example, Chen *et al.* demonstrated that cytokines present in murine bone marrow-derived MSC-CM stimulated macrophages, endothelial migration, and wound healing in BALB/c mice [37]. Similarly, another study showed that the paracrine effects of MSCs accelerated the regeneration of endogenous stem cells and ameliorated obstruction-induced overactive bladders [38]. Kim *et al.* reported that soluble intracellular adhesion molecule-1 secreted by human umbilical cord blood-derived MSCs reduced the number of amyloid-β plaques, which can cause Alzheimer’s disease [39]. In our previous study, cytokine array analysis revealed that hDSPC-CM contains higher levels of multiple growth factors, including basic fibroblast growth factor, hepatocyte growth factor, insulin-like growth factor-binding protein-1 and -2, insulin-like growth factor, and vascular endothelial growth factor, compared with non-hDSPC-CM [19]. Thus, we speculate that the beneficial effects of hDSPC-CM on senescent fibroblasts may be mediated by hDSPC-secreted growth factors.

According to previous reports, cell cycle regulators and DNA damage response pathways are strongly implicated in the onset of senescence [1,2,3,5,6,20]. For example, increased p53 phosphorylation forces cells into a state of premature senescence [2,3,4]; as cells enter G0, p21, a well-known target gene of p53, increases in a p53-dependent manner. Thus, we investigated the expression of these proteins and demonstrated that hDSPC-CM significantly down-regulated p53 phosphorylation as well as p21 expression (Figure 2C).

ROS are also considered to be major factors contributing to the aging process. Metabolic dysfunction and exogenous stress can generate excessive ROS levels [22,23,24]. Our data demonstrated that hDSPC-CM decreased H_2_O_2_ levels in senescent fibroblasts (Figure 4). To investigate the underlying mechanism to explain this phenomenon, we further examined the expression of several antioxidant enzymes, including members of the SOD family and catalase. According to previous reports, SOD1 is located in the cytoplasm, SOD2 is sequestered to the mitochondria, and SOD3 is secreted into the extracellular matrix [40,41]. Interestingly, we observed that only SOD2 expression was specifically enhanced in senescent fibroblasts cultured with hDSPC-CM compared with control and non-hDSPC-CM (Figure 5), implying that increased ROS production by senescent fibroblasts is mainly generated in the mitochondria [40,41].

**Figure 6:**
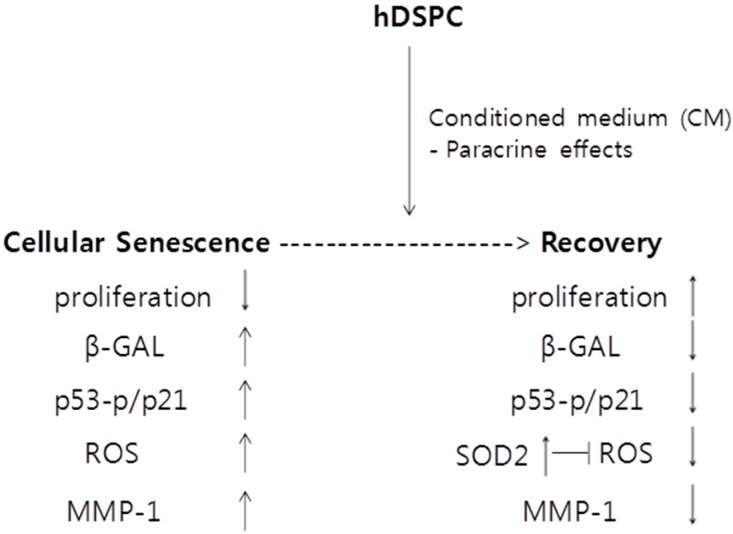
A schematic representation of the mechanism contributing to the beneficial effects of hDSPC-CM on senescent human dermal fibroblasts. ↑ means increase, whereas ↓ means decrease.

In conclusion, we suggest that hDSPC-CM ameliorates fibroblast senescence in a paracrine manner (Figure 6). Thus, the factors secreted by hDSPCs represent potential therapeutics to promote skin regeneration.

## 4. Experimental Section

### 4.1. Preparation of hDSPC-CM

HDFs (Lonza, Basel, Switzerland) were cultured in Dulbecco’s modified eagle medium (DMEM) (Lonza) containing 10% fetal bovine serum (FBS), 100 U/mL penicillin, and 100 μg/mL streptomycin (Life Sciences, St. Petersburg, FL, USA) at 5% CO_2_ and 37 °C. Fibroblasts were used within three passages.

To enrich hDSPCs that are present in only small amounts in fibroblasts, collagen type IV (Sigma-Aldrich, St. Louis, MO, USA)-coated dishes were prepared by coating 150-mm tissue culture dishes with collagen type IV (20 mL, 20 μg/mL) overnight at 4 °C. We observed that >50% of the HDFs adhered to the collagen type IV-coated dishes after 10 min of incubation. Therefore, we gradually reduced the adherence time in 1-min increments. Cells were not to adhere to dishes in less than 4 min. Ultimately, an incubation time of 12 h was selected because most of the cells could adhere to the dishes during this period. Thus, the cells were separated based on their ability to adhere to the plates within 4–5 min (hDSPCs) or within 12 h (non-hDSPCs) at 37 °C. About 5%–10% of the total fibroblasts adhered to the dishes within 4–5 min of incubation [17,18].

To obtain the CM, hDSPCs (1 × 10^5^ cells/mL) were cultured in a Hydrocell™ dish (Nunc, Penfield, NY, USA) with serum-free DMEM. After 48 h in culture, hDSPC-CM was collected, centrifuged at 300× *g* for 5 min, and filtered with a 0.22-μm syringe filter (PALL) [19].

### 4.2. Cell Culture and CM Treatment

HDFs derived from adult skin (Cat. no. CC-2511) were purchased from Lonza and cultured in DMEM supplemented with 10% FBS (Lonza), 100 U/mL penicillin, and 100 μg/mL streptomycin (Life Sciences) at 5% CO_2_ and 37 °C. Senescent fibroblasts were established as previously described [20,21].

For CM treatment, 3 × 10^5^ cells were seeded onto 60-mm dishes in the CM, and DMEM with 50% non-hDSPC-CM or 50% hDSPC-CM was added to the cultures for the indicated times. All CMs were changed every 24 h.

### 4.3. Immunofluorescence Staining for Ki67

Senescent fibroblasts were incubated with 50% non-hDSPC-CM or hDSPC-CM for 24 h. Following CM treatment, the cells were fixed with 4% formaldehyde (Sigma-Aldrich) and then blocked with 1% bovine serum albumin (Sigma-Aldrich). The cells were then incubated with mouse monoclonal anti-Ki67 antibody (Abcam, Cambridge, MA, USA) for 12 h at 4 °C, followed by Alexa-488-conjugated secondary antibody (Invitrogen, Carlsbad, CA, USA) for 1.5 h at room temperature. The nuclei were stained with 5 mg/mL DAPI (Sigma) for 5 min. Samples were washed with phosphate-buffered saline with Tween-20 after each step. Slide-mounted samples were imaged with an EVOS fluorescence microscope (Advanced Microscopy Group, Mill Creek, WA, USA). Three images per dish were collected, and Ki67-positive cells were counted.

### 4.4. Cell Proliferation Assay

Senescent fibroblasts were seeded onto 96-well culture plates and treated with non-hDSPC-CM or hDSPC-CM at the indicated dilutions for 24 h. Following each CM treatment, 10 μL of Cell Counting Kit-8 (Sigma-Aldrich) was added to each well. After incubating for 1 h, absorbance at 450 nm was measured.

### 4.5. SA-β-Gal Assay

SA-β-Gal activity was detected using a senescence detection kit (Abcam) following the manufacturer’s protocol. Briefly, senescent fibroblasts were treated with 50% non-hDSPC-CM or hDSPC-CM for 72 h. The cells were then fixed and incubated at 37 °C with a staining solution containing 1 mg/mL of 5-bromo-3-chloro-4-indolyl β-d-galactoside (X-gal) for 12 h. Five images per dish were collected, and the blue cells were counted.

### 4.6. Western Blot Analysis

Senescent fibroblasts were incubated with 50% non-hDSPC-CM or hDSPC-CM for 72 h. The cells were lysed in RIPA buffer (Millipore, Billerica, MA, USA) supplemented with phosphatase inhibitors (Sigma-Aldrich) and proteinase inhibitors (Roche, Indianapolis, IN, USA). Protein lysates (20 μg per lane) were run on 4%–12% Bis-Tris gels (Life Technologies, Carlsbad, CA, USA), transferred to polyvinylidene fluoride membranes (Roche), and analyzed by Western blotting. β-actin levels were measured in the samples as a control for protein loading using rabbit polyclonal anti-β-actin antibody (Santa Cruz Biotechnology, Santa Cruz, CA, USA). Phosphorylated and total p53 and p21 (Waf1/Cip1) levels were detected by rabbit polyclonal anti-phospho-p53 (serine 397), mouse monoclonal anti-p53, and rabbit monoclonal anti-p21, respectively (Cell Signaling Technology, Boston, MA, USA).

### 4.7. RNA Isolation and Real-Time RT-PCR

Total RNA was extracted using TRI Reagent^®^ (Invitrogen) and quantified on a NanoDrop spectrophotometer (Thermo Scientific, Lafayette, CO, USA). Reverse transcription of 4 μg total RNA was performed using ReverTra Ace reverse transcriptase (Toyobo, Osaka, Japan), and the reaction was terminated by adding Tris-EDTA buffer (pH 8.0) to 200 μL of the cDNA (2 μg) solution. Real-time RT-PCR was performed using a 7500 Fast Real-Time PCR System (Applied Biosystems, Foster City, CA, USA), according to the manufacturer’s instructions. Briefly, 20 μL of PCR mixture contained 10 μL 2 × TaqMan^®^ universal PCR Master Mix, 50 ng cDNA, and 1 μL 20 × TaqMan^®^ Gene Expression assay reagent (Applied Biosystems). cDNA samples were analyzed to determine the expression levels of *COL1A1*, Hs00164004_m1; *COL3A1*, Hs00943809_m1; *MMP1*, Hs00899658_m1; *TIMP1*, Hs00171558_m1; *SOD1*, Hs00533490_m1; *SOD2*, Hs00167309_m1; and *SOD3*, Hs00156308_m1. Human *GAPDH* (43333764F) (Applied Biosystems) was used for normalizing the variation in cDNA quantities from different samples.

### 4.8. H_2_O_2_ Production Measurements

H_2_O_2_ released from senescent HDFs was measured using the Amplex Red Hydrogen Peroxide/Peroxidase Assay Kit (Life Technologies), according to the manufacturer’s instructions. Senescent HDFs were treated with non hDSPC-CM or hDSPC-CM. The CM (50 μL) of each sample was harvested and incubated with 100 μL reaction solution containing 100 μM Amplex Red reagent and 0.2 U/mL horseradish peroxidase for 10 min. The fluorescence of each sample was measured at 590 nm emission following excitation at 560 nm using a Gemini XPS microplate reader (Molecular Device, Sunnyvale, CA, USA).

### 4.9. Statistical Analysis

Statistical analysis was performed using SPSS software (IBM Corporation, Armonk, NY, USA). An unpaired Student’s *t*-test or one*-*way analysis of variance was used as indicated in the figure legends. *p*-values less than 0.05 were considered statistically significant.

## 5. Conclusions

For the first time, we demonstrated that CM derived from hDSPCs exhibits various anti-aging effects on senescent HDFs. hDSPC-CM not only reduced SA-β-gal levels but also decreased p53 phosphorylation and p21 expression in senescent HDFs. In addition, hDSPC-CM restored the expression of a major structural protein, collagen type I, and reduced H_2_O_2_ levels in senescent HDFs. In conclusion, we suggest that hDSPC-CM can be clinically used as a potential therapeutic agent for improving human aged skin.
